# Effectiveness of psychoeducation in reducing sickness absence and improving mental health in individuals at risk of having a mental disorder: a randomised controlled trial

**DOI:** 10.1186/s12889-015-2087-5

**Published:** 2015-08-08

**Authors:** Pernille Pedersen, Hans Jørgen Søgaard, Merete Labriola, Ellen A. Nohr, Chris Jensen

**Affiliations:** Psychiatric Research Unit West, Regional Psychiatric Services West, Central Denmark Region, Gl. Landevej 49, 7400 Herning, Denmark; Institute of Clinical Medicine, University of Aarhus, Aarhus, Denmark; Public Health and Quality Improvement, Central Denmark Region, Aarhus, Denmark; Section of Clinical Social Medicine and Rehabilitation, School of Public Health, University of Aarhus, Aarhus, Denmark; Institute of Clinical Research, University of Southern Denmark, Odense, Denmark; Department of Public Health and General Practice, Norwegian University of Science and Technology, NTNU, Trondheim, Norway; National Centre for Occupational Rehabilitation, Rauland, Norway

**Keywords:** Sickness absence, Psychoeducation, Mental health, Return to work, Psychological symptoms, Mental health-related quality of life, Locus of control

## Abstract

**Background:**

The aim of this study was to evaluate the effect of psychoeducation on return to work as an adjunct to standard case management in individuals on sick leave at risk of having a mental disorder. The participants could have different diagnoses but were all at risk of having a mental disorder.

**Methods:**

Between 2012 and 2014, 430 participants on sick leave were randomly allocated to either an intervention or control group. The psychoeducation consisted of 2-h sessions once a week for 6 weeks. The sessions focused on stress and work life and was based on problem-solving techniques and coping strategies. The main outcome, the relative risk (RR) of a full return to work based on register data from the job centres, was determined during the first 3 and 6 months after participation in the psychoeducation programme. At baseline and at 3 and 6 months after the intervention, the participants received a questionnaire on psychological symptoms, mental health-related quality of life, and locus of control.

**Results:**

During the first 6 months after inclusion, the two groups had almost the same RR of a full return to work (RR:0.97, 95 % CI: 0.78;1.21), but during the first 3 months, the individuals in the intervention group had a significantly higher risk of not having fully returned to work (RR:0.68, 95 % CI:0.47;0.98). The individuals in the intervention group who had participated in at least four of the six psychoeducational sessions returned to work considerably slower at both time points than did the control group. The intervention did not decrease the level of psychological symptoms or improve mental health-related quality of life; however, individuals in the intervention group improved their scores on internal locus of control at both 3 and 6 months.

**Conclusion:**

Offering psychoeducation to individuals on sick leave at risk of having a mental disorder had no influence on the chance of a full return to work during the first 6 months; however, it did result in a higher relative risk of not returning to work after 3 months. Therefore, we do not recommend offering psychoeducation in this form to facilitate return to work.

**Trial registration:**

Clinical Trial.gov NCT01637363. Registered 6 July 2012.

## Background

Common mental disorders, such as adjustment disorders, depression, anxiety, and somatoform disorders, are highly prevalent in the working population [1–3]. In the Western countries, mental disorders are a main cause of sick leave [4–6], estimated to be involved in more than half of all individuals on long-term sickness absence [7]. Sickness absence due to mental health problems has a considerable societal impact, in addition to the individual consequences in the form of reduced quality of life as well as a reduced functional ability and workability [8]. The high prevalence of individuals on sick leave with a mental disorder calls for stronger emphasis to meet the needs of this group. Moreover, studies have shown that mental disorders are likely to be underestimated because of both under-recognition and under-reporting of mental disorders as a reason for sickness absence [9–11]. Thus, it has been recommended that the social workers in the Danish municipal case management centres (job centres) screen and identify individuals with mental health problems in order to be able to offer a tailored return to work (RTW) intervention [12]. Early identification and intervention are assumed to shorten the length of spells of sickness absence, hasten RTW [11], and result in a better prognosis for the mental disorder [8, 12]. Early action seems especially important as long-term sickness absence is a predictor of future disability pension [13].

In relation to interventions and treatment in Denmark, the introduction of shared care models has been suggested to facilitate a better connection between case management in the social sector and specialist mental healthcare [12]. Some studies have included interventions by a specialist in mental health care with the aim of reducing symptoms and enhance participants’ coping skills in relation to work [14–16]. Psychoeducation (PE) is a simple therapy offered to individuals with mental disorders in the healthcare systems and in primary care settings [17–19] and gives the patients a theoretical and practical approach towards understanding and coping with the consequences of the disorder [20]. It has been assumed that PE can modify an individual’s perception of themselves and their future by giving information, correcting dysfunctional thoughts, and thereby assisting adaption. Moreover, it has been assumed that when PE provides individuals with information about symptoms, they might find these experiences to be less disturbing [21].

Overall, PE has proven to be able to improve clinical outcomes in patients with a psychiatric disorder [22–24], besides increasing participation in pleasant activities, social interaction [25], self-esteem [25, 26], and the frequency of seeking social support [25]. These acquired competences will presumably be helpful in the RTW process. To the best of our knowledge, PE has not previously been used specifically as an offer to individuals on sickness absence at risk of having a mental disorder [8].

The aim of this study was to evaluate the effect of PE targeted specifically to facilitate RTW as an adjunct to standard case management in individuals on sick leave at risk of having a mental disorder. The first consultation at the job centre between the social worker and the individual on sick leave is often based on self-reported diagnosis. But as mental disorders are likely to be underestimated, it seems important to screen and identify individuals at risk of having a mental disorder.

It was hypothesized that individuals who participated in the PE programme would have shorter periods of sickness absence than would a control group, and furthermore, fewer psychological symptoms, and improved mental health-related quality of life and internal locus of control.

## Methods

### Study design, procedure, and participants

**Fig. 1:**
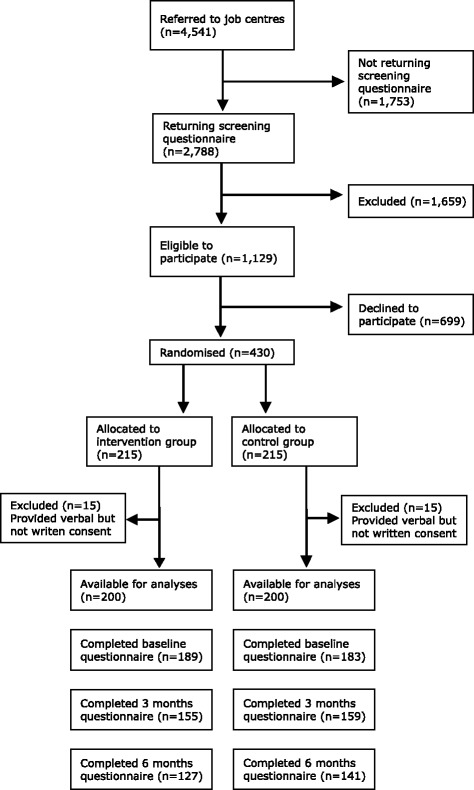
Flow chart of the study

A randomised controlled trial (RCT) was conducted among individuals on sick leave in four municipalities in the Western part of Denmark. Between September 2012 and January 2014, 4541 individuals who had been on sick leave for 4–8 weeks received by mail information about the study and a screening questionnaire. Individuals were included in the study if they were between 18 and 64 year of age, on sick leave from work or unemployment, and had a SCL-8 AD score ≥5 [27]. SCL-8 AD was used to identify individuals at risk of having a mental disorder. Individuals who did not communicate in Danish, had been on sick leave due to mental health problems for more than 3 consecutive months during the preceding year, were pregnant, or had a supported job/were in job training/in rehabilitation/had retired were excluded (*n* = 1659, see Flowchart Fig. 1).

Eligible individuals were contacted by phone and given information about the study. If they agreed to participate in the study, they were randomised (block size 4) based on a computerised random number generator into the intervention group or the control group. Subsequently, they were mailed information about their allocation and a consent form to fill out and return. This allocation procedure was chosen to avoid delay in starting the PE programme because of late arrival of written consent forms. Participants who were randomised to the intervention group based on oral consent but failed to provide written consent were excluded from further data collection (*n* = 30).

All study participants were on sick leave and thus, obliged by law to participate in consultations with the social workers at the job centres. The social workers provide the usual social services at the job centres, and in collaboration with the individuals on sickness absence benefit, they assess whether the individuals are ready to RTW. The social workers were not informed about the allocation of the participants. However, they could have been aware of it, which could have influenced the RTW outcome. Therefore, the social workers were asked to guess the allocation of the randomisation for 176 randomly selected participants about 3 months after the randomisation.

Sickness absence data were assessed from registers in the job centres. A research assistant and two social workers collected the administrative data on RTW, but they were blinded for study allocation. At baseline and at 3 and 6 months of follow-up, the participants received a questionnaire to assess secondary outcomes.

Participation was voluntary, and the study was notified to and registered by the Danish Data Protection Agency (http://www.datatilsynet.dk). According to the Danish National Committee on Biomedical Research Ethics, the intervention did not need ethical approval as it did not include biomedical research. The study is registered at Clinical Trials.gov (NCT01637363).

A more thorough description of the method of the study and the ethical considerations has previously been published in a protocol paper [28].

### Treatments

#### Psychoeducation

The intervention group was offered PE in group sessions, and an early start of the intervention had a high priority. Thus, the participants were offered PE shortly after they had orally accepted to participate, which was also the reason that open groups were applied. The intervention consisted of six 2-h sessions once a week and was held at two different locations. The open groups ran continually throughout the study period, and each session was conducted about nine times at each location. Participants who were unable to join a specific session had the opportunity to join the session next time. The number of participants in each session was on average 7 (SD 3.8), varying from 1–18.

The intervention was conducted and taught by four psychiatric nurses who were experienced in psychoeducation, a psychologist, a social worker, a physiotherapist, and a person previously on sick leave due to mental health problems. The psychiatric nurses were accustomed to practising PE, and one of the psychiatric nurses was present at each session.

The sessions focused on stress and work life and consisted of a mixture of didactic lectures and group discussions based on problem-solving techniques and coping strategies. The purpose was to impart knowledge about psychiatric conditions in order to provide individuals on sick leave with qualifications to understand and improve their own situation. The focus was, to a high extent, on the general discomfort in everyday life caused by the symptoms and in particular on handling a job and to a less extent on diagnosis. The intervention followed structured slides that had been developed by the teachers and had the following content: information about the symptoms of adjustment disorders, depression, anxiety, and somatoform disorders; information about specific, useful, cognitive tools in regard to the barriers and difficulties they might experience when re-entering the work force; the interaction between physical exercise and mental health; the sickness absence legislation and the implication of it; experiences from a person previously on sick leave due to mental health problems. Furthermore, the relatives of the participants were invited to hear about mental health problems and sickness absence to further the understanding of their relatives’ situation [28].

### Usual care

All the participants received usual care offered by the job centres, which typically comprises fitness workout, stress and pain management, and a gradual RTW. The Danish sickness benefit law does not specify which kind of activities should be available. Consequently, a large variation exists across municipalities [12]. Because of the study’s natural setting, all participants were free to engage in any other treatment as well.

### Outcome measures

RTW was operationalised as not receiving sickness benefits and measured by register data from the municipalities’ job centres.

### Primary outcome

*Time to full RTW* was defined as the period (in days) between randomisation and not receiving any sickness benefits for at least 4 weeks without partial or full sickness absence recurrence.

### Secondary outcome

*Time to first RTW* was defined as the period (in days) between randomisation and to partial or full-time RTW without partial or full sickness absence recurrence. Thus, the participants could still receive partial sickness benefits.

*Psychological symptoms* of psychopathologic status were assessed with the Symptom Checklist-90-Revised (SCL-90-R) [29], a 90-item self-rating instrument for assessing the discomfort, as described in each item, experienced during the past 7 days. The discomfort is assessed on a 5-point rating scale ranging from “not at all” (0) to “extremely” (4). The instrument is divided into nine scales; however, only six of these were of interest in this study: somatization, obsessive-compulsive, interpersonal sensitivity, depression, anxiety, and phobic anxiety. The Danish version of the questionnaire was used [30].

*Mental health-related quality of life* was assessed by the 36-item Short Form Health Survey (SF-36) [31], a self-administered health survey with 36 items grouped into eight scales. Only the four scales related to mental health were of interest in this study: vitality, social functioning, role limitations due to emotional problems, and mental health. A high score indicates a better level of functioning (range 0–100). Furthermore, the question “In general, would you say your health is…” was included. Answers were dichotomized as good (response options excellent, very good and good), and poor (response options fair and poor). The Danish version of the instrument was used [32].

*Health locus of control* was assessed by The Multidimensional Health Locus of Control (MHLC) scale Form C [33]. It can be defined as the degree to which individuals believe that their health is controlled by internal or external factors. The Form C is condition-specific and can be used when studying individuals with an existing health/medical condition. Participants were asked to consider the condition responsible for the sickness absence. Form C consists of four subscales: “doctors” and “other people”, each with three items, and “chance” and “internal”, each with six items. For each item, a Likert scale ranging from 1 to 6 was applied (1 representing “strongly disagree” and 6 representing “strongly agree”).

A translation of the questionnaire into Danish was done for the present study, and it was tested in a pilot study.

### Covariates

The screening questionnaire provided information on gender, age, the highest level of education, and employment. Moreover, the individuals were asked to state their own reasons for the sickness absence, a reason which had not necessarily been confirmed by a doctor. They could report several of the following reasons: anxiety, depression, other mental illness, stress and burnout, psychosocial working environment, musculoskeletal disorders and also cardiovascular or lung diseases, infection, chronic/diffuse pain, cancer, abdominal illness, personal problems, which were categorized as other reasons (Table 1). Furthermore, they were asked to report their recovery expectations, which were their own estimation in percentage (0–100 %) of the probability of not being on sick leave after 6 months.

**Table 1:** Baseline characteristics of the study population

Variable	Intervention group (*n* = 215)		Control group (*n* = 215)	
	Mean/median/n	SD/IQR/ %	Mean/median/n	SD/IQR/ %
Gender (female), n	154	49.8	155	50.2
Age (years), mean	43.5	10.0	43.9	9.9
Length of sickness absence until randomization (days), mean	56.4	22.1	57.2	18.3
Highest level of education, n				
Primary school or high school	40	18.6	52	24.2
<3 years	105	48.8	90	41.9
>3 years	70	32.6	73	34.0
Employment, n				
Student	16	7.4	6	2.8
Unemployed	37	17.2	33	15.4
Unskilled worker	33	15.3	34	15.8
Basic skilled worker	29	13.5	26	12.1
Wage-earning and salaried employees	86	40.0	104	48.4
Self-employed	11	5.1	10	4.7
Don’t know / not available	3	1.4	2	0.9
Reason for sickness absence, n ^a^				
Anxiety	54	25.1	46	21.4
Depression	85	39.5	91	42.3
Other mental illness	12	5.6	8	3.7
Stress and burnout	122	56.7	115	53.5
Psychosocial working environment	51	23.7	49	22.8
Musculoskeletal disorders	43	20.0	53	24.7
Other reasons	79	36.7	74	34.4
Number of symptoms (SCL-8 AD), mean	9.8	2.3	9.8	2.4
Recovery expectations, n				
0–50 % or don’t know/not available	81	37.7	90	41.9
60–90 %	67	31.2	53	24.7
100 %	67	31.2	72	33.5
Sick-leave, n				
Full-time sick leave	214^*^	99.5	208^*^	96.7
Part-time sick leave	1	0.5	7	3.3
Locus of control, median^b^				
Internal	22.0^*^	18.0–26.0	20.0^*^	15.0–25.0
Chance	14.0	11.0–18.0	14.5	11.0–18.0
Doctor	12.0	10.0–14.0.	12.0	10.0–14.0
Other people	11.0	9.0–13.0	11.0	8.0–13.0
Psychological symptoms, median^b^				
Somatization	1.1	0.6–1.7	1.2	0.7–1.8
Anxiety	1.2	0.6–1.8	1.2	0.6–1.8
Interpersonal sensitivity	1.2	0.8–1.9	1.3	0.8–2.0
Depression	1.8	1.2–2.5	1.9	1.2–2.6
Phobic anxiety	0.4	0.1–0.9	0.4	0.1–1.1
Obsessive compulsive	1.6	1.0–2.3	1.7	1.0–2.3
Health-related QoL, median^b^				
Vitality (VT)	30.0	20.0–40.0	30.0	15.0–45.0
Social functioning (SF)	62.5	37.5–87.5	62.5	37.5–87.5
Role limitations due to emotional problems (RE)	33.3	0.0–33.3	0.0	0.0–33.3
Mental health	48.0	36.0–60.0	48.0	36.0–56.0
General health, n				
Poor	101	54.0	101	56.1
Good	86	46.0	79	43.9

*IQR* interquartile range, *SD* standard deviation

^*^ *P*-value <0.05

^a^Several reasons were possible for each individual

^b^Completed by 189 in the intervention group and 183 in the control group

The records from the job centres were used to retrieve information on whether the participants were fully or partially on sick leave.

In the questionnaire 3 months after randomisation, the participants were asked if they had participated in RTW activities (usual care) arranged by the job centres and co-interventions such as treatment by a general practitioner (GP), a psychologist, or a psychiatrist.

### Statistical analysis

To evaluate the effectiveness of PE compared to usual care, the rates of RTW during the first 3 and 6 months after randomisation were compared by means of the pseudo values method [34, 35]. The relative risk (RR) of returning to work in the intervention group was compared to that in the control group. Furthermore, the cumulative incidence proportion (CIP) was calculated for the specific time points to show the percentages of individuals in each group who had returned to work. Analyses were performed for both full RTW and first RTW. Participants were right-censored if their sickness absence benefits had been suspended because they had moved to another municipality, the duration of sickness absence had reached the time limit (52 weeks during the previous 18 months), or the job centres reported that the individual did not cooperate. Individuals who had died or had been transferred to other benefits such as early retirement or supported job were treated as competing risk. However, in the analyses for first RTW, individuals who started in supported employment were considered as having returned to work as they were working a few hours a week. A total of 11 individuals were right-censored during the first 6 months, and one experienced a competing risk event. For the outcome full RTW, data for the first 12 months of follow-up were shown in a cumulative incidence probability plot adjusted for competing risk.

Analyses were performed according to the intention-to-treat principle. Moreover, per-protocol analyses were performed by comparing participants in the control group with participants in the intervention group who had attended at least four of the six sessions.

The differences in scores on psychological symptoms, mental health-related quality of life (QoL) and locus of control (LoC) between the groups were analysed at 3 and 6 months. As many of the items or subscales did not have a normal distribution, the Wilcoxon-Mann-Whitney test was used. No adjustment for the scores from the baseline questionnaire was performed, as some participants first filled out the questionnaire after they had started the intervention. Response rates to specific items were not below 94.5 %. Only complete cases were included in the analyses.

Participants were compared with those who were eligible but declined participation.

All point estimates are presented with 95 % confidence intervals. A two-sided probability of *p <* 0.05 was considered statistically significant for the primary outcome and *p* < 0.005 for the secondary outcomes. STATA/IC 11.2 (StataCorp LC, College Station, TX, USA) was used for all statistical analyses.

## Results

### Participants

A total of 1129 individuals were eligible for participation, and 430 (38 %) agreed to participate and were randomised to the intervention group (*n* = 215) or the control group (*n* = 215) (Fig. 1). The characteristics of the 430 individuals are given in Table 1. The groups did not differ with respect to background variables; however, individuals in the intervention group had a higher score on internal LoC and slightly more individuals from that group were on full-time sick leave. Mental health problems as reason for sickness absence were almost the same in the two groups. In the intervention group, 25 % reported anxiety, 40 % reported depression and 57 % reported stress and burn out as reason for the absence, while the frequencies were 21 %, 42 %, and 54 %, respectively, in the control group. The 4 job centres were of different sizes and thus did not include the same number of participants. The distributions of participants were 43 %, 28 %, 14 % and 15 %, respectively, from each job centre and were evenly distributed between the two groups, *p* = 0.67.

Compared to those who declined to participate, participants were more likely to be women, to have an education, to be on sick leave due to anxiety, depression, stress or burnout, or to have complained of a poor psychosocial working environment. They were less often on sick leave due to cancer or musculoskeletal disorders. Moreover, they had a higher SCL-8 AD score and lower recovery expectations.

A total of 15 individuals from the intervention group and 15 individuals from the control group provided only verbal consent and were excluded from the study. The questionnaires were completed by 189 (95 %) and 183 (92 %) at baseline, 155 (78 %) and 159 (80 %) at 3 months, and 127 (64 %) and 141 (71 %) at 6 months by participants from the intervention group and control group, respectively. There was no difference between those who completed the 6-months questionnaire and those who did not in relation to age, gender, education, and SCL-8 AD score.

### Psychoeducation

Not all individuals from the intervention group participated in all of the PE sessions. A total of 176 individuals (88 %) participated in at least one of the sessions, 132 (66 %) participated four to six times, 44 (22 %) participated one to three times, and 24 (12 %) did not show up. Furthermore, 74 participants brought a relative.

On average, participation in the first session took place 16 days after randomisation (range: 2–91 days) and 73 days after the first day of sickness absence (range: 22–134 days).

The individuals who participated four to six times were on average older than those who participated less than four times (45.3 vs 40.2 years, *p* < 0.001). The different participation levels were not related to gender, education, or SCL-8 AD score.

### Participation in usual care and co-interventions

No differences between groups were found for participation in usual care or co-interventions. A total of 99 (64 %) individuals in the intervention group and 107 (69 %) in the control group had received treatment for their mental condition 3 months after the intervention. In the intervention group, those who had received treatment had received it from a GP (72 (73 %)), a psychologist (78 (79 %)), a psychiatrist (14 (14 %)), or elsewhere (22 (22 %)). The corresponding numbers for individuals in the control group were 80 (75 %), 74 (69 %), 8 (7 %), and 18 (17 %), respectively. No significantly differences were found between the groups.

A total of 65 (42 %) vs. 57 (36 %) from the intervention group and control group, respectively, had participated in activities offered by the job centres. In both the intervention group and control group, the most frequent activities attended were physical training/exercise (44 (68 %) and 32 (56 %), respectively) and mindfulness therapy (12 (18 %) and 18 (32 %), respectively).

### Sick leave

**Fig. 2:**
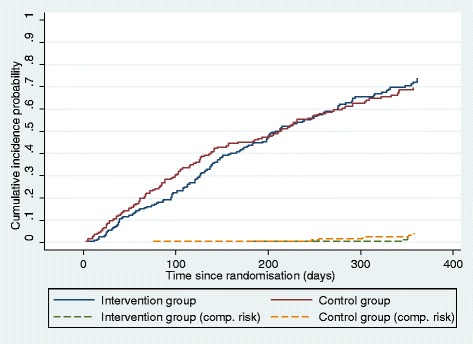
Cumulative incidence probability of full work resumption and competing risk from randomisation until 1 year after. Intervention group (*n* = 200) and control group (*n* = 200)

The two groups had almost the same relative chance of full RTW during the first 6 months after the randomisation (RR 0.97, Table 2, Fig. 2). Nearly half of the participants in both groups had fully returned to work at that time. However, during the first 3 months, the individuals in the intervention group had a statistically significantly higher risk of not having fully returned to work, as only 19 % of the individuals had returned compared to 28 % in the control group.

**Table 2:** Chance of return to work according to participation in psychoeducation

	Control group *n* = 200	Intervention group	Intervention group
		Intention-to-treat *n* = 200	Per-protocol *n* = 132
Full RTW^a^			
3 mo	28 (22;35)	19 (14;25)	11 (5;16)
CIP % (95 % CI)	1 (ref)	0.68 (0.47;0.98)	0.38 (0.22;0.65)
RR (95 % CI)			
6 mo	45 (38;52)	44 (37;51)	40 (31;48)
CIP % (95 % CI)	1 (ref)	0.97 (0.78;1.21)	0.89 (0.68;1.15)
RR (95 % CI)			
First RTW^b^			
3 mo	38 (31;44)	31 (25;38)	26 (19;34)
CIP % (95 % CI)	1 (ref)	0.83 (0.63;1.09)	0.69 (0.49;0.97)
RR (95 % CI)			
6 mo	52 (45;59)	49 (42;56)	46 (38;55)
CIP % (95 % CI)	1 (ref)	0.94 (0.77;1.14)	0.88 (0.70;1.11)
RR (95 % CI)			

CIP (Cumulative Incidence Proportion) shows the percentages of individuals having returned to work

^a^Competing risk: death or other benefits such as early retirement or supported job

^b^Competing risk: death or other benefits (except supported job)

From randomisation to 12 months, the intervention group had a RR of 1.06 (95 % CI: 0.92–1.22) for having fully returned to work compared to the control group. A total of 74 % and 70 % had returned to work in the intervention group and control group, respectively (results not shown in Table).

In relation to first RTW, no significant differences were found between the groups at either time points; however, trends were similar to what was seen for full RTW.

The individuals in the intervention group who had participated in at least four of the six psychoeducational sessions returned to work (both full RTW and first RTW) considerably later at both time points than was the case in the control group (Table 2).

### Mental health

No significant differences in psychological symptoms were found between the two groups at any time point (Table 3). The participants in the intervention group reported a significantly higher score on internal LoC at both time points, but no differences were found for the other three LoC variables. Neither did we observe any differences between the groups for vitality, social functioning, role limitations due to emotional problems, or mental health at either time point.

**Table 3:** Mental health at 3 and 6 months according to participation in psychoeducation

		3 months					6 months				
		Intervention *n* = 152–155		Control *n* = 157–159			Intervention *n* = 124–127		Control *n* = 133–141		
		Median	IQR	Median	IQR	*p*-value^*^	Median	IQR	Median	IQR	*p*-value^*^
Psychological symptoms	Somatisation	0.7	0.3–1.1	0.8	0.3–1.3	0.09	0.6	0.3–1.0	0.7	0.3–1.3	0.20
	Anxiety	0.6	0.2–1.1	0.8	0.3–1.3	0.04	0.4	0.2–0.9	0.6	0.2–1.2	0.09
	Interpersonal sensitivity	0.7	0.4–1.2	0.9	0.4–1.6	0.08	0.7	0.2–1.2	1.0	0.3–1.4	0.10
	Depression	1.0	0.5–1.7	1.3	0.7–2.3	0.02	0.8	0.5–1.5	1.1	0.5–1.9	0.19
	Phobic anxiety	0.1	0.0–0.6	0.1	0.0–0.6	0.29	0.1	0.0–0.4	0.1	0.0–0.4	0.27
	Obsessive-compulsive	1.0	0.5–1.8	1.2	0.7–2.0	0.12	0.8	0.4–1.5	1.0	0.6–1.7	0.05
Locus of control	Internal LOC	23.0	19.0–28.0	20.0	16.0–25.0	<0.001	24.0	19.5–28.0	21.0	16.0–25.0	<0.001
	Chance	14.0	11.0–18.0	14.5	11.0–18.0	0.23	14.0	9.0–18.0	14.0	10.0–18.0	0.43
	Doctors	12.0	10.0–14.0	12.0	10.0–14.0	0.50	12.0	9.0–13.0	12.0	10.0–13.0	0.76
	Other people	11.0	9.0–13.0	11.0	8.0–13.0	0.39	10.0	8.0–12.0	10.0	8.0–12.0	0.88
Mental health related QoL	Vitality	45.0	30.0–60.0	45.0	25.0–60.0	0.32	50.0	30.0–65.0	50.0	25.0–65.0	0.54
	Social functioning	75.0	62.5–100.0	87.5	62.5–100.0	0.98	87.5	75.0–100.0	87.5	62.5–100.0	0.58
	Role limitations due to emotional problems	66.7	33.3–100.0	66.7	0.0–100.0	0.33	66.7	33.3–100.0	66.7	33.3–100.0	0.71
	Mental health	64.0	52.0–76.0	60.0	44.0–76.0	0.04	68.0	56.0–80.0	68.0	52.0–78.0	0.41

*IQR* interquartile range

^*^Wilcoxon-Mann–Whitney- test, significance level <0.005

A total of 94 (61 %) participants in the intervention group and 82 (52 %) participants in the control group reported a good general health at 3 months. At 6 months, the numbers were 80 (63 %) and 85 (63 %). No statistically significant difference was found at either time point (*p* = 0.12 and *p* = 0.93, respectively).

The social workers who assessed readiness to RTW and allocated job centre activities to the participants provided a guess regarding allocation group for 96 (55 %) randomly selected participants. They were able to guess the allocation correctly for two-thirds of the participants in the control group, but only guessed half of the allocations correctly for the participants in the intervention group.

## Discussion

### Main findings

The aim of the study was to evaluate the effect of PE in individuals at risk of having a mental disorder. Participating in the PE sessions had no influence on the chance of full RTW during the first 6 months, but during the first 3 months, participants in the intervention group had a significantly higher risk of not having fully returned to work. The same pattern was seen for the outcome first RTW; however, no significant difference was observed during the first 3 months. The risk of not returning to work during the first 3 months was highest for individuals who had participated in four to six sessions compared to the control group.

The intervention did not decrease the level of symptoms of depression or anxiety or any other of the psychological symptoms. It did not improve mental health related QoL; however, individuals in the intervention group improved their scores on internal LoC at both 3 and 6 months.

### Interpretation of outcomes

The significantly higher risk in the intervention group of not returning to work during the first 3 months might be due to an ambition to complete the PE programme before they went back to work. It is plausible since individuals who participated four to six times had an even higher risk of not going back to work compared to the risk of all participants in the intervention group. For all individuals allocated to the intervention group, the chance for first RTW was not significantly lower than in the control group. This may be because they had been able to attend the course while working part time. As part of usual care, individuals from both groups participated in other courses arranged by the job centres, e.g. psychology sessions and mindfulness therapy. It was not examined whether participating in those courses resulted in a higher risk of not returning to work. However, it might not be the participation in PE or other courses in itself that delayed RTW, but the fact that they participated in a research project and, therefore, wanted to finish the intervention even though they were ready to RTW. It has been presumed that participating in an intervention programme for several weeks may obstruct the natural RTW and, hence, introduce a negative effect [36]. Another explanation for delayed RTW could be that the course made them more aware of their mental health symptoms, and therefore, they felt worse and postponed RTW. However, participants in the intervention group did not score higher on mental health symptoms after the intervention compared to the control group.

If PE or course participation may, in general, result in a higher risk of not returning to work, it is important to be aware of when implementing interventions. Maybe the risk is more pronounced when the intervention is offered close to the start of the sickness absence period. Most workers will return to work rapidly within the first months after reporting sick [37, 38]. Participating in interventions at an early stage could therefore prolong RTW. In individuals on sick leave due to low back pain, the optimum time window for the start of an effective structured intervention has been suggested to be approximately 8 to 12 weeks after start of the sickness absence [36]. Our intervention was, on average, provided 7–8 weeks after the start of sickness absence. However, it could be questioned whether the intervention started too early because participants in the control group returned to work significantly earlier than did the intervention group during the first 3 months after the intervention was initiated.

### Psychoeducation

This specific type of PE was not effective in facilitating RTW and improving mental health. This could be due to the intervention not being specific and tailored to the participants’ individual needs. PE is usually applied to a group of patients with one specific diagnosis [17]. In this study, the participants could suffer from sub-clinical as well as clinical depression, anxiety, and somatoform disorder besides feeling distressed. Broad inclusion criteria were applied because we believed that the topics that were taught and discussed in the psychoeducation sessions would be relevant for sick-listed individuals with different mental health problems. Another reason for the broad inclusion criteria was to test an intervention that could be implemented by the social workers in the job centres without asking medical doctors for specific diagnostic information.

Another reason for not finding an effect could be the open groups, which were used in order to offer the intervention as rapidly as possible, as it has been shown to be important for RTW outcome [11]. This, however, resulted in a lack of continuity in the PE because participants had not all attended the same previous sessions. Furthermore, the participants were not well connected socially since they only took part in a few sessions together. This also limited their opportunity to exchange experiences with other participants.

Another reason for not finding an effect could be that the sessions might have been based too much on lectures and too little on discussions. Thus, the content of the sessions might not have been sufficiently aimed at the participants’ own challenges. It is possible that homework would have helped the participants to work with the topics and make them part of their daily lives. We did not measured how well they used what they had been taught.

Furthermore, the course may have focused too much on mental health and not enough on RTW. The nurses were not accustomed to working with individuals on sick leave or giving advice on RTW issues; however the physiotherapist, the social worker, and the psychologist were. Finally, PE was given in addition to the standard offers to individuals on sick leave in Denmark. Thus about 40 % of the individuals participated in activities offered by the job centres, and about 65 % received treatment for their mental health, mostly from a GP or a psychologist. Moreover, the social workers encouraged the participants to resume to part time work partially, which may facilitate RTW [39]; however, the effect in individuals with mental disorders is inconsistent [38, 40].

In the analysis of the effect of the intervention in this study, the content of usual care must be considered. The effectiveness of the intervention, in this case PE, is a relative measure and depended on the effect in the usual care group, which may have been effective in itself.

#### Strength and limitations

The major strength of this study was the randomised design and the large group of participants. Register data were used to measure RTW, which is preferable compared to self-reported data in regard to receiving more accurate information on the sick leave period [41].

The social workers were not sufficiently blinded for the allocation and were able to correctly identify two-thirds of the individuals in the control group, which could introduce confounding. It is possible that they could have let participants in the control group return to work earlier than those in the intervention group.

The intervention was offered at an early stage in the sickness absence period. As a result, participants were randomised before they had given written consent. This could introduce possible risk of bias, but it did not seem to have affected the final results.

Thus the participants knew their allocation before they provided written consent; however, this did not seem to influence the relative participation rates because the same number of individuals from each group dropped out of the study after randomisation. The internal validity of the study does not seem to have been threatened because no differences were found between the dropouts in the two groups. Reasons for dropping out of the study and reasons for not attending the PE session as intended were not collected.

Some participants completed the baseline questionnaire after they had started the intervention. Analyses were not adjusted for baseline score as this could introduce information bias. However, scores on symptoms of depression and anxiety (SCL-90-R) at baseline and the score on SCL-8 AD were similar for the two groups. The SCL-8 AD consists of items on symptoms of depression and anxiety and was completed by participants before they knew about their allocation. Moreover, the scores on the remaining baseline questions seemed to be similar between the two groups. However, the significantly higher score on internal LoC in the intervention group at both 3 and 6 months might be explained by the difference that was already present at baseline.

#### Generalisation

Effectiveness of RCTs depends on the context in which they are conducted. Effectiveness in RCTs in the field of RTW will differ due to heterogeneity in populations, characteristics of the workers and workplaces, and differences in the social system [42]. The study was performed in individuals on sick leave in a Danish setting, and all participants received the standard care from the job centres and health care system.

The present study was conducted in collaboration with the job centres, because the goal was to assess the effect of the intervention as it would work in a realistic setting. The participants were included based on a simple screening instrument (SCL-8 AD). Thus, considerable variation in reasons for sickness absence, symptoms, and diagnoses was allowed.

One-third of the eligible individuals participated in the study. The study population consisted of more women than men and of individuals who were intermediate to highly educated, on sick leave due to mental health problems, and had low recovery expectations, which is similar to another Danish study [43]. It is possible that those accepting to participate were more eager to return to work compared to those not accepting to participate. If the last two-thirds had participated, it is likely that the results would have been different from those in the present study.

PE was taught by different health professionals, which circumvents ascribing the effect to have been due to the influence of a single person.

## Conclusion

Offering PE to individuals on sick leave at risk of having a mental disorder had no influence on the chance of full RTW during the first 6 months; however, it did result in a higher risk of not returning to work during the first 3 months after randomisation. Moreover, it did not decrease the level of psychological symptoms or improve mental health-related quality of life and internal locus of control. Based on this study, offering PE in this form in a municipal job centre setting in order to facilitate RTW cannot be recommended.
